# Impact of nonalcoholic fatty liver disease on the response to pegylated interferon-α therapy in patients with chronic hepatitis B

**DOI:** 10.1128/spectrum.03424-25

**Published:** 2026-07-06

**Authors:** Huiqing Liang, Yizhi Xie, Yan Dai, Xiaorong Yang, Xiaoting Zheng, Jiaen Yang, Yaoyu Liu, Chuncheng Wu, Siyan Chen, Manying Zhang, Hongli Zhuang, Li Lin, Shaodong Chen

**Affiliations:** 1 Hepatology Unit, Xiamen Hospital of Traditional Chinese Medicine, Xiamen, Fujian, China; 2 School of Clinical Medicine, Fujian University of Traditional Chinese Medicine 47858, Fuzhou, Fujian, China; 3 Department of Traditional Chinese Medicine, No.1 Hospital, Xiamen University 599493 , Xiamen, Fujian, China; 4 Department of Traditional Chinese Medicine, School of Medicine, Xiamen University 599493 , Xiamen, Fujian, China; University of Manitoba, Winnipeg, Manitoba, Canada

**Keywords:** chronic hepatitis B, nonalcoholic fatty liver disease, pegylated interferon-alpha, HBsAg clearance, predictors

## Abstract

Hepatitis B surface antigen (HBsAg) clearance is regarded as the optimal therapeutic endpoint in patients with chronic hepatitis B (CHB). Pegylated interferon-α (PegIFNα) is currently one of the most effective strategies for achieving this goal. However, the impact of concurrent nonalcoholic fatty liver disease (NAFLD) on the PegIFNα treatment response in CHB patients remains controversial. The aim of this study was to investigate the impact of NAFLD on the antiviral efficacy of PegIFNα in patients with CHB and identify baseline predictors of HBsAg clearance. In this retrospective cohort study, 201 CHB patients receiving PegIFNα monotherapy or PegIFNα in combination with nucleos(t)ide analogs were enrolled and stratified into CHB-only (*n* = 125) and CHB + NAFLD (*n* = 76) groups based on the presence of hepatic steatosis. Baseline clinical, virological (including HBsAg levels), and histological characteristics were collected and compared. Cumulative HBsAg clearance, biochemical response, and virological response rates were assessed. Two Cox proportional hazards regression models were constructed to identify independent predictors of HBsAg clearance through univariable and multivariable analyses. No significant difference was observed in the 48-week cumulative HBsAg clearance rate between the CHB + NAFLD and CHB-only groups (18.42% vs 23.2%, *P* = 0.41), nor in the virological response rates. However, the biochemical response rate was significantly lower in the CHB + NAFLD group (13.79% vs 22.03%, *P* = 0.042). Multivariate Cox regression analysis identified baseline HBsAg levels (Model 2: HR = 0.36, 95% CI: 0.26–0.51, *P* < 0.001) and baseline hepatitis B virus (HBV) DNA levels (Model 2: HR = 0.79, 95% CI: 0.63–1.00, *P* = 0.049) as independent predictors of HBsAg clearance, while NAFLD comorbidity was not independently associated with HBsAg clearance (*P* = 0.58). In CHB patients receiving PegIFNα-based therapy, concurrent NAFLD did not significantly impair HBsAg clearance rates (18.42% vs 23.2% in week 48). However, it was associated with a reduced biochemical response rate. Baseline levels of HBsAg and HBV DNA served as independent predictors of HBsAg clearance.

**IMPORTANCE:**

Chronic hepatitis B (CHB) and nonalcoholic fatty liver disease (NAFLD) are two of the most common liver diseases worldwide, and their coexistence is increasingly frequent due to the rising prevalence of metabolic disorders. Achieving hepatitis B surface antigen clearance is a key therapeutic goal, and pegylated interferon-α (PegIFNα) remains one of the most effective treatments to achieve it. However, it remains unclear whether the presence of NAFLD influences the response to PegIFNα therapy. The study found that NAFLD does not impair the clearance or suppression of hepatitis B virus, but it is associated with delayed normalization of biochemical markers. Whether the hepatitis B surface antigen becomes negative depends mainly on baseline viral levels, rather than the presence of NAFLD. These findings provide important clinical insights for the management of patients with both CHB and NAFLD.

## INTRODUCTION

Chronic hepatitis B virus (HBV) infection remains a major global public health threat and is a leading cause of cirrhosis, hepatocellular carcinoma (HCC), and hepatitis-related mortality. According to the World Health Organization, the global prevalence of the HBsAg is approximately 3.2%, corresponding to an estimated 254 million individuals living with chronic HBV infection (1).

Nonalcoholic fatty liver disease (NAFLD), the hepatic manifestation of metabolic syndrome, has emerged as one of the most prevalent chronic liver diseases worldwide. Meta-analyses indicate a global NAFLD prevalence of 25.24%, with rates reaching 27.4% in Asia and exceeding 30% in the Middle East and South America (2). As metabolic disorders become increasingly commonplace, the co-occurrence of chronic HBV and NAFLD is rising. Epidemiological studies estimate that 29.6%–46.8% of individuals with chronic HBV infection are affected by NAFLD (3), indicating a substantial overlap between the two diseases in HBV-endemic regions.

Pegylated interferon-α (PegIFNα) remains a cornerstone therapeutic option in the management of chronic hepatitis B (CHB), especially for patients aiming to achieve a functional cure. Compared to nucleos(t)ide analogs (NAs), PegIFNα offers the unique advantage of inducing immune-mediated viral suppression and HBsAg seroclearance, making it a critical strategy for securing long-term clinical benefits (4, 5).

However, the impact of NAFLD on the treatment outcomes of CHB patients receiving PegIFNα remains controversial. Some studies suggest that concurrent NAFLD may promote HBsAg seroclearance and suppress HBV replication (6–8). Conversely, other studies report no significant effect on HBsAg clearance and even caution that NAFLD may increase the risk of HCC by accelerating hepatic fibrosis (9, 10). Moreover, existing research presents inconsistent findings regarding the effect of NAFLD on biochemical and virological responses to antiviral therapy, particularly with PegIFNα. While some investigations report that NAFLD impairs biochemical and virological responses (11, 12), others observe no substantial impact (7, 13). These discrepancies may largely be attributed to heterogeneity in study designs, baseline viral characteristics, NAFLD severity, and treatment regimens.

Given the increasing clinical intersection of NAFLD and CHB, alongside the persistent uncertainties surrounding treatment responses, further investigation is warranted. This study aims to evaluate the clinical outcomes of CHB patients with concurrent NAFLD undergoing PegIFNα-based therapy, with a specific focus on HBsAg seroclearance. Additionally, it seeks to identify key baseline predictors of antiviral treatment response, thereby helping to inform individualized management strategies for this complex patient population.

## MATERIALS AND METHODS

### Study subjects

This retrospective study included 201 patients with CHB enrolled at the Center of Liver Diseases, Xiamen Hospital of Traditional Chinese Medicine, between December 2021 and June 2024. All participants were treated with a PegIFNα-based antiviral regimen—either PegIFNα-2b (Pegbing, Xiamen Amoytop Biotech Co., Ltd.) monotherapy or PegIFNα-2b in combination with NAs. Patients were required to have complete medical records from their initial hospital admission. Based on liver biopsy results, the cohort was stratified into two groups: those with CHB only (*n* = 125) and those with concurrent CHB and NAFLD (*n* = 76).

Eligible participants were adults (≥18 years) meeting the established diagnostic criteria for CHB (and NAFLD, where applicable), with comprehensive clinical, laboratory, and follow-up data. Exclusion criteria were as follows: coinfection with hepatitis A, C, or D viruses, or human immunodeficiency virus; a history of alcohol-related liver disease, drug-induced liver injury, or autoimmune liver disease; and a diagnosis of HCC or other malignancies.

### Clinical assessments and study endpoints

All enrolled patients underwent systematic clinical and laboratory evaluations every 12 weeks from baseline through week 48. Laboratory assessments included HBV DNA quantification via high-sensitivity fluorescence PCR, HBV serological markers (HBsAg, HBeAg, and anti-HBe) measured using an electrochemiluminescence immunoassay, and liver function parameters (e.g., alanine aminotransferase [ALT]).

Ultrasound-guided liver biopsy was performed, and histological evaluations were conducted by experienced pathologists. Hepatic inflammatory activity and fibrosis were staged according to the Ishak scoring system, while hepatocellular steatosis was quantified by the proportion of affected hepatocytes.

The core outcome measures and their definitions were as follows: HBsAg clearance (HBsAg <0.05 IU/mL), biochemical response (ALT <40 U/L), virological response (HBV DNA <20 IU/mL), HBeAg loss (HBeAg <1 S/CO), and HBeAg seroconversion (HBeAg <1 S/CO with concurrent anti-HBe seropositivity <1 S/CO).

### Statistical analysis

Statistical analyses were performed using R software version 4.3.3 (R Foundation for Statistical Computing). Baseline characteristics were compared between the two groups using independent samples *t*-tests for normally distributed continuous variables and Mann-Whitney *U* tests for non-normally distributed variables. Categorical variables were evaluated using the chi-square or Fisher’s exact tests, as appropriate; cumulative response rates were estimated and visualized using Kaplan-Meier curves, with intergroup differences assessed via the log-rank test.

To identify independent predictors of HBsAg seroclearance, Cox proportional hazards regression analyses were conducted. Two multivariable analysis modeling strategies were applied to ensure analytical robustness. Model 1 incorporated variables that demonstrated significance in the univariate analysis (*P* ≤ 0.05), while Model 2 utilized variables selected through least absolute shrinkage and selection operator (LASSO) regression. The primary outcome was HBsAg seroclearance, and a two-sided *P* value of <0.05 was considered statistically significant.

## RESULTS

### Baseline characteristics

This study retrospectively identified 201 patients with complete data, with a median age of 37 years (interquartile range: 32–43 years). Among them, 125 were diagnosed with CHB alone, and 76 had concurrent CHB and NAFLD. The treatment regimens received during the study period consisted of either PegIFNα monotherapy (*n* = 80) or PegIFNα combined with NAs (*n* = 121), with comparable allocation between the two groups. Additional baseline characteristics are summarized in Table 1. Univariate analysis revealed statistically significant intergroup differences in gender, alcohol consumption history, body mass index (BMI), steatosis grade, total cholesterol (TC), low-density lipoprotein (LDL), gamma-glutamyl transpeptidase (GGT), and uric acid (UA) levels (Table 1).

**TABLE 1:** Baseline characteristics of patients with vs without NAFLD^*a*^

Variable	Overall (*n* = 201)	CHB (*n* = 125)	CHB + NAFLD (*n* = 76)	*P* value^*b*^
Age (years)	37 (32–43)	36 (31–43)	40 (33–43)	0.170
Gender, *n* (%)				0.001
Male	146 (72.6)	81 (64.8)	65 (85.5)	
Female	55 (27.4)	44 (35.2)	11 (14.5)	
Family history of hepatitis B, *n* (%)				0.990
No	148 (73.6)	92 (73.6)	56 (73.7)	
Yes	53 (26.4)	33 (26.4)	20 (26.3)	
Alcohol consumption history, *n* (%)				0.004
No	172 (85.6)	114 (91.2)	58 (76.3)	
Yes	29 (14.4)	11 (8.8)	18 (23.7)	
BMI (kg/m^2^)	23.3 (21.4–25.4)	22.0 (20.0–23.9)	25.1 (23.5–27.8)	<0.001
Treatment regimen, *n* (%)				0.207
PegIFNα	80 (39.8)	54 (43.2)	26 (34.2)	
PegIFNα + NAs	121 (60.2)	71 (56.8)	50 (65.8)	
Liver fibrosis stage, *n* (%)				0.898
<2	157 (78.1)	98 (78.4)	59 (77.6)	
≥2	44 (21.9)	27 (21.6)	17 (22.4)	
Liver inflammation grade, *n* (%)				0.161
<2	45 (22.4)	32 (25.6)	13 (17.1)	
≥2	156 (77.6)	93 (74.4)	63 (82.9)	
HBV DNA (log_10_ IU/mL)	4.87 (2.65–7.55)	5.06 (2.61–7.55)	4.74 (2.70–7.56)	0.985
HBsAg (log_10_ IU/mL)	3.45 (2.61–4.24)	3.45 (2.61–4.27)	3.40 (2.61–4.19)	0.808
HBeAg (C.O.I)	0 (0–409)	0 (0–309)	0 (0–606)	0.877
Anti-HBe (C.O.I)	0 (0–14)	1 (0–13)	0 (0–17)	0.373
ALT (U/L)	57 (29–102)	58 (25–102)	56 (36–98)	0.538
AST (U/L)	34 (23–61)	36 (22–63)	33 (25–52)	0.969
UA (mmol/L)	356 (303–415)	346 (289–387)	385 (319–459)	<0.001
TC (mmol/L)	4.70 (4.20–5.50)	4.60 (4.10–5.20)	5.05 (4.20–5.90)	0.011
TG (mmol/L)	1.06 (0.77–1.51)	0.94 (0.71–1.26)	1.40 (0.92–1.79)	<0.001
HDL (mmol/L)	1.30 (1.07–1.53)	1.36 (1.16–1.55)	1.17 (1.01–1.39)	<0.001
LDL (mmol/L)	2.91 (2.37–3.61)	2.82 (2.23–3.32)	3.30 (2.58–3.95)	0.001
GLU (mmol/L)	5.00 (4.70–5.20)	4.90 (4.70–5.20)	5.07 (4.75–5.40)	0.077
TBIL (mmol/L)	14 (11–19)	14 (11–19)	15 (10–19)	0.973
DBIL (mmol/L)	5.00 (3.40–7.20)	5.20 (3.70–7.20)	4.70 (3.20–7.00)	0.185
ALP (U/L)	70 (59–83)	69 (59–79)	75 (59–92)	0.040
GGT (U/L)	31 (19–55)	28 (17–52)	42 (25–70)	0.002
Albumin (g/L)	45.0 (42.0–47.0)	44.0 (42.0–46.0)	45.0 (42.0–47.0)	0.272
Globulin (g/L)	29.0 (27.0–33.0)	29.0 (27.0–33.0)	30.0 (27.0–32.0)	0.773

^*a*^ Continuous variables are presented as medians (interquartile ranges).

^*b*^ *P *values are derived from the Wilcoxon rank sum test, Pearson’s Chi-squared test, or Fisher’s exact test, as appropriate.

### Outcome measures

#### HBsAg clearance

**Fig 1:**
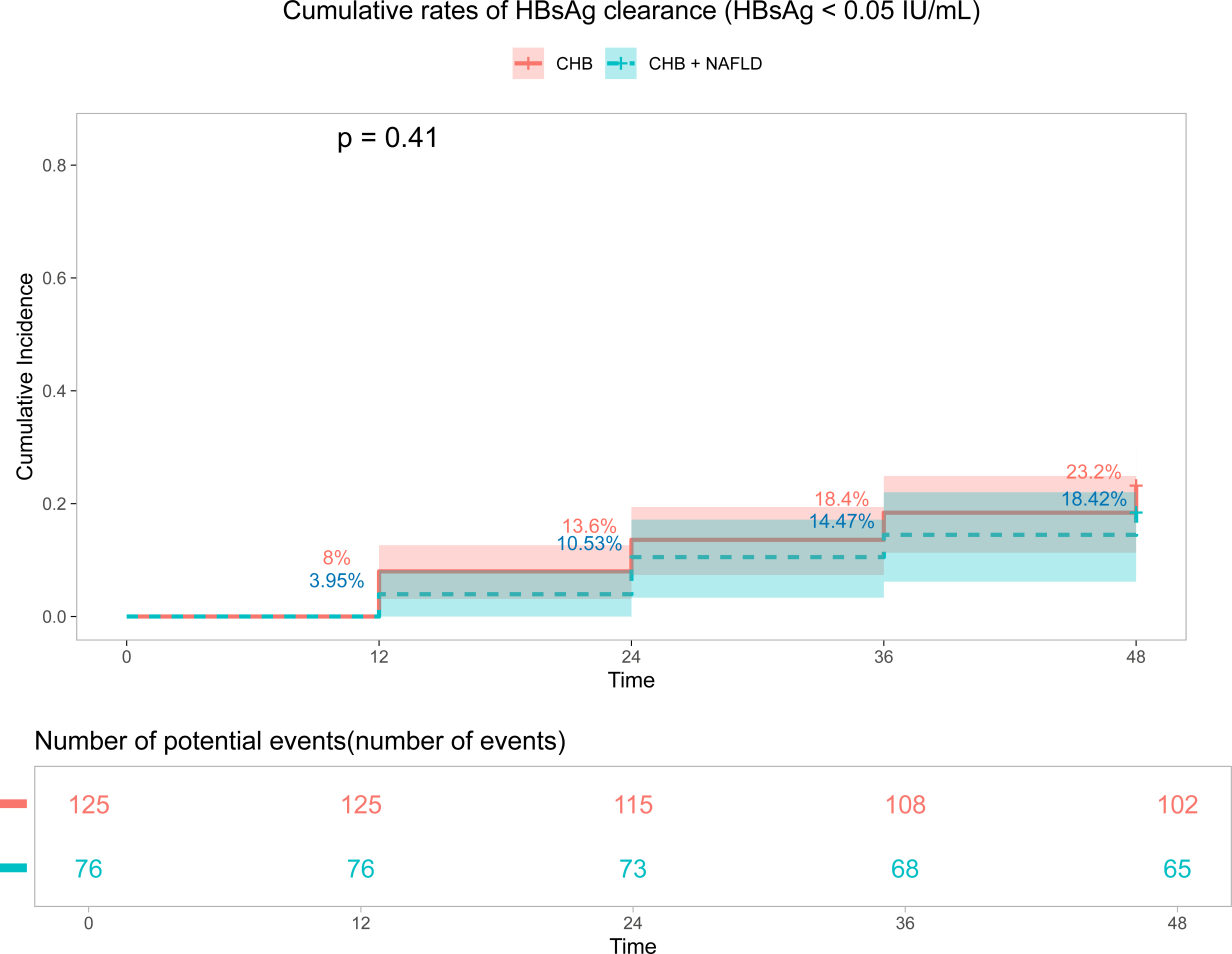
Kaplan-Meier curve for HBsAg clearance rates.

The Kaplan-Meier analysis revealed that both groups exhibited a steady upward trend in HBsAg clearance over the treatment period, with no statistically significant divergence between them (Fig. 1). Notably, the cumulative HBsAg clearance rates achieved at each time point were relatively high, highlighting the favorable efficacy of PegIFNα-based therapy in this cohort.

At week 12, the clearance rates were 3.95% in the CHB + NAFLD group and 8.0% in the CHB-only group; by week 24, these increased to 10.53% vs 13.6%, and by week 36, 14.47% vs 18.4%. By week 48, the cumulative HBsAg clearance rate reached 18.42% in the CHB + NAFLD group and 23.2% in the CHB-only group—remarkably high given the baseline HBsAg levels of the enrolled patients, reflecting the potent antiviral and immunomodulatory effects of PegIFNα. No significant differences were identified at any time point (*P* > 0.05).

#### Biochemical response (ALT)

**Fig 2:**
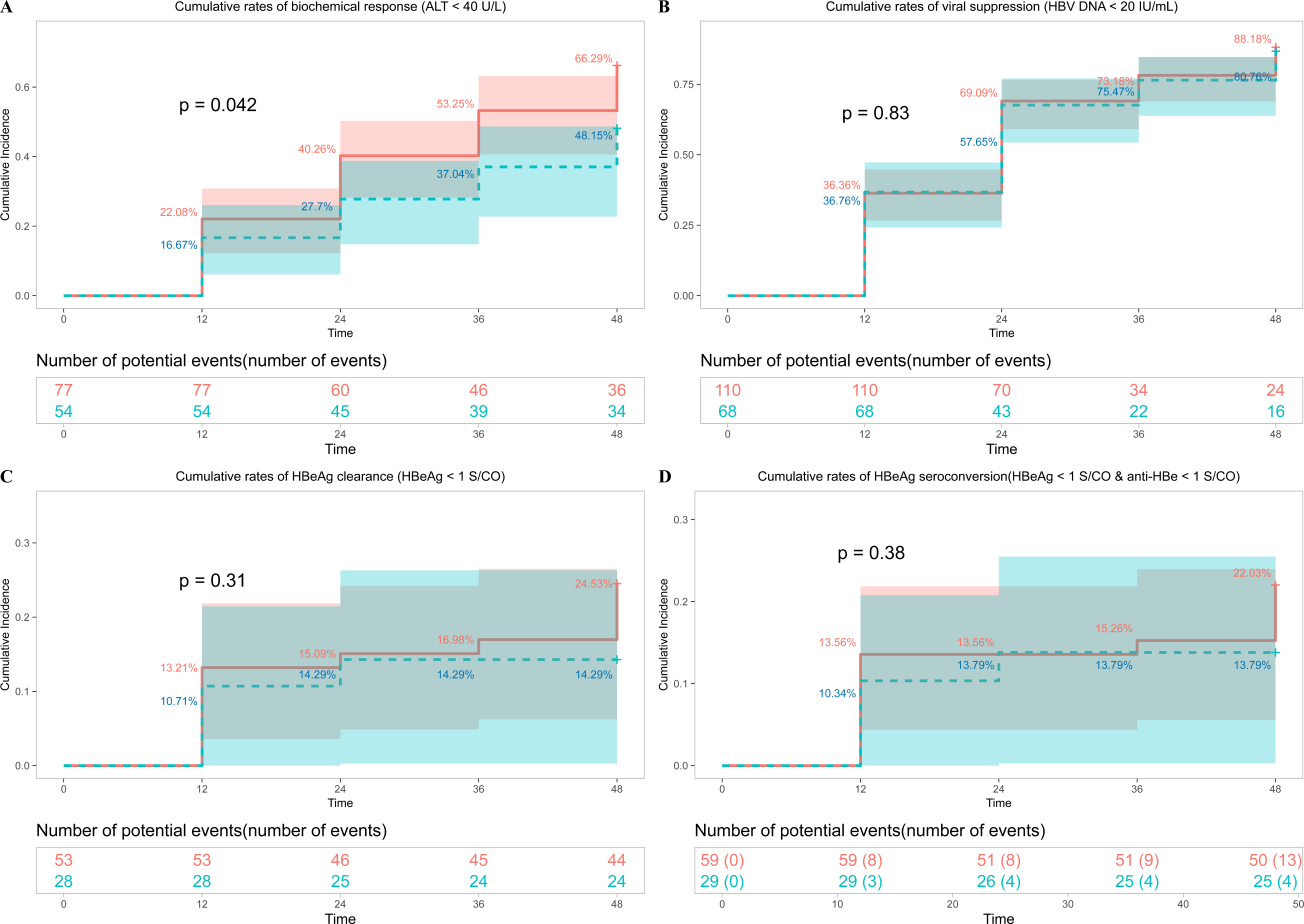
Kaplan-Meier curves for biochemical, viral, and HBeAg responses. (**A**) Biochemical response; (**B**) viral suppression; (**C**) HBeAg clearance; and (**D**) HBeAg seroconversion.

Patients with concomitant NAFLD exhibited a modestly reduced biochemical response rate compared to those without NAFLD (*P* = 0.042). Cumulative ALT normalization rates at 12, 24, 36, and 48 weeks were 10.34% vs 13.56%, 13.79% vs 13.56%, 13.79% vs 15.25%, and 13.79% vs 22.03%, respectively (Fig. 2A).

#### Virological response (HBV DNA)

No significant difference was observed in virological response rates between patients with and without NAFLD (*P* = 0.83). At 12, 24, 36, and 48 weeks, the respective viral suppression rates were 16.67% vs 22.08%, 27.78% vs 40.26%, 37.04% vs 53.25%, and 48.15% vs 66.23%, respectively (Fig. 2B).

#### HBeAg loss and seroconversion

There was no significant difference in HBeAg loss rates between patients with and without NAFLD (*P* = 0.31). HBeAg loss rates at 12, 24, 36, and 48 weeks were 10.71% vs 13.21%; 14.29% vs 15.09%; 14.29% vs 16.98%; and 14.29% vs 24.53%, respectively (Fig. 2C).

Likewise, no significant difference in HBeAg seroconversion rates was observed between CHB patients with and without NAFLD (*P* = 0.38). The respective seroconversion rates at 12, 24, 36, and 48 weeks were 10.34% vs 13.56%, 13.79% vs 13.56%, 13.79% vs 15.25%, and 13.79% vs 22.03% (Fig. 2D).

### Analysis of factors influencing HBsAg clearance

#### Univariate analysis

In the univariate analysis, several baseline factors were significantly associated with HBsAg clearance, including a family history of HBV infection (HR = 0.41, 95% CI: 0.17–0.97, *P* = 0.043), baseline HBV DNA level (HR = 0.58, 95% CI: 0.49–0.69, *P* < 0.001), baseline HBsAg level (HR = 0.36, 95% CI: 0.30–0.44, *P* < 0.001), baseline HBeAg negativity (HR = 17.1, 95% CI: 4.14–70.8, *P* < 0.001), and baseline anti-HBe positivity (HR = 6.59, 95% CI: 2.59–16.7, *P* < 0.001). Additional significant variables included baseline ALT, AST, DBIL, GGT, and albumin (Table 2).

**TABLE 2:** Univariate analysis of factors influencing HBsAg clearance

Characteristics	Univariate analysis	
	HR (95% CI)^*a*^	*P* value
Age (years)	1.02 (0.99–1.06)	0.18
Gender, *n* (%)		
Male	–^*b*^	
Female	1.22 (0.63–2.33)	0.56
Treatment regimen, *n* (%)		
PegIFNα	–	
PegIFNα + NAs	0.59 (0.33–1.08)	0.087
Family history of hepatitis B, *n* (%)		
No	–	
Yes	0.41 (0.17–0.97)	0.043
Alcohol consumption history, *n* (%)		
No	–	
Yes	0.40 (0.12–1.30)	0.13
Liver fibrosis stage		
<2	–	
≥2	1.06 (0.52–2.15)	0.87
Liver inflammation grade		
<2	–	
≥2	0.37 (0.20–0.69)	0.002
HBV DNA (log_10_ IU/mL)	0.58 (0.49–0.69)	<0.001
HBsAg (log_10_ IU/mL)	0.36 (0.30–0.44)	<0.001
HBeAg		
Positive	–	
Negative	17.1 (4.14–70.8)	<0.001
Anti-HBe		
Negative	–	
Positive	6.59 (2.59–16.7)	<0.001
ALT (U/L)	0.99 (0.98–1.00)	0.009
AST (U/L)	0.98 (0.97–1.00)	0.015
UA (mmol/L)	1.00 (1.00–1.00)	0.73
TC (mmol/L)	0.84 (0.60–1.17)	0.29
TG (mmol/L)	0.88 (0.59–1.30)	0.51
HDL (mmol/L)	1.34 (0.53–3.34)	0.54
LDL (mmol/L)	0.97 (0.69–1.37)	0.88
GLU (mmol/L)	0.99 (0.96–1.03)	0.73
TBIL (mmol/L)	0.97 (0.93–1.02)	0.25
DBIL (mmol/L)	0.88 (0.78–1.00)	0.050
ALP (U/L)	0.99 (0.97–1.00)	0.13
GGT (U/L)	0.99 (0.97–1.00)	0.041
Albumin (g/L)	1.06 (1.01–1.10)	0.008
Globulin (g/L)	1.01 (0.98–1.05)	0.51

^*a*^ HR, hazard ratio; CI, confidence interval.

^*b*^ "–" denotes the reference group for the categorical variables in the Cox regression analysis. The hazard ratio (HR) for the reference group is fixed at 1.00; therefore, no confidence interval or *P* value is calculated.

#### Multivariate analysis

To account for the baseline imbalances observed between the CHB + NAFLD and CHB groups (Table 1), we performed multivariable Cox regression analyses with sex, BMI category, UA, TC, and TG as covariates to determine the independent effect of NAFLD.

In multivariable Model 1, which included only those variables with *P* < 0.05 in univariate analysis, baseline HBsAg was identified as an independent predictor (HR = 0.29, 95% CI: 0.19–0.45, *P* < 0.001).

In Model 2, constructed using LASSO regression, both baseline HBsAg (HR = 0.36, 95% CI: 0.26–0.51, *P* < 0.001) and baseline HBV DNA (HR = 0.79, 95% CI: 0.63–1.00, *P* = 0.049) emerged as significant independent predictors (Table 3).

**TABLE 3:** Multivariate analysis of factors influencing HBsAg clearance

Characteristics	Model 1		Model 2	
	HR (95% CI)^*a*^	*P* value	HR (95% CI)^*a*^	*P* value
Age (years)	0.99 (0.94–1.04)	0.61		
Gender, *n* (%)				
Male				
Female	0.83 (0.31–2.17)	0.70	1.02 (0.44–2.39)	0.96
Treatment regimens				
PegIFNα				
PegIFNα + NAs				
Family history of hepatitis B, *n* (%)	–^*b*^			
No				
Yes	1.05 (0.38–2.90)	0.92		
Family history of liver cancer	–			
No				
Yes				
Alcohol consumption history, *n* (%)				
No				
Yes				
BMI (kg/m^2^)				
Underweight, [18.5, 23)				
Normal, <18.5	1.62 (0.44–5.99)	0.47	1.19 (0.39–3.60)	0.76
Overweight, ≥23	0.64 (0.30–1.34)	0.24	0.56 (0.27–1.16)	0.12
Liver fibrosis stage, *n* (%)				
<2				
≥2	2.01 (0.81–5.01)	0.13		
Liver inflammation grade, *n* (%)				
<2				
≥2				
Steatosis grade				
<1				
≥1				
Presence of fatty liver				
CHB				
CHB + NAFLD				
HBV DNA (log_10_IU/mL)	0.98 (0.71–1.34)	0.88	0.79 (0.63–1.00)	0.049
HBsAg (log_10_ IU/mL)	0.29 (0.19–0.45)	<0.001	0.36 (0.26–0.51)	<0.001
HBeAg (C.O.I)				
Positive	–			
Negative	3.02 (0.35–25.8)	0.31		
Anti-HBe (C.O.I)				
Positive	–			
Negative	0.56 (0.14–2.21)	0.41		
ALT (U/L)	1.00 (1.00–1.01)	0.59		
AST (U/L)				
UA (mmol/L)	1.00 (1.00–1.01)	0.36	1.00 (1.00–1.01)	0.41
TC (mmol/L)	0.63 (0.43–0.91)	0.014	0.72 (0.50–1.02)	0.067
TG (mmol/L)	0.59 (0.30–1.14)	0.12	0.60 (0.35–1.04)	0.068
HDL (mmol/L)				
LDL (mmol/L)				
GLU (mmol/L)				
TBIL (mmol/L)				
DBIL (mmol/L)	0.82 (0.70–0.97)	0.020		
ALP(U/L)				
GGT(U/L)	0.99 (0.97–1.01)	0.28		
Albumin (g/L)	1.05 (0.99–1.11)	0.14		
Globulin (g/L)				

^*a*^ HR, hazard ratio; CI, confidence interval.

^*b*^ "–" denotes the reference group for the categorical variables in the Cox regression analysis. The hazard ratio (HR) for the reference group is fixed at 1.00; therefore, no confidence interval or *P* value is calculated.

#### Subgroup analysis

To further substantiate our findings, a subgroup analysis was performed comparing the baseline characteristics of patients who achieved HBsAg clearance in the CHB + NAFLD group (*n* = 14) and the CHB-only group (*n* = 29). Differences were noted in BMI, hepatic steatosis severity, uric acid, and alkaline phosphatase levels, which are inherently aligned with NAFLD diagnostic criteria. However, no significant differences were observed in other baseline variables. Notably, baseline HBsAg levels were comparable between the CHB + NAFLD and CHB groups (*P* = 0.808), reinforcing the notion that baseline HBsAg remains the predominant predictor of HBsAg clearance in this cohort (Table 4).

**TABLE 4:** Baseline characteristics of patients achieving HBsAg clearance^*a*^

Variable	Overall (*n* = 43)	CHB (*n* = 29)	CHB + NAFLD (*n* = 14)	*P* value^*b*^
Age (years)	39 (33–47)	39 (32–47)	39 (34–43)	0.631
Gender, *n* (%)				0.164
Male	30 (69.8)	18 (62.1)	12 (85.7)	
Female	13 (30.2)	11 (37.9)	2 (14.3)	
Family history of hepatitis B, *n* (%)				>0.999
No	37 (86.0)	25 (86.2)	12 (85.7)	
Yes	6 (14.0)	4 (13.8)	2 (14.3)	
Alcohol consumption history, *n* (%)				0.029
No	40 (93.0)	29 (100.0)	11 (78.6)	
Yes	3 (7.0)	0 (0.0)	3 (21.4)	
BMI (kg/m^2^)	22.9 (21.1–25.3)	21.8 (20.1–24.5)	24.6 (23.7–26.5)	0.016
Treatment regimen, *n* (%)				0.449
PegIFNα	22 (51.2)	16 (55.2)	6 (42.9)	
PegIFNα + NAs	21 (48.8)	13 (44.8)	8 (57.1)	
Liver fibrosis stage, *n* (%)				0.704
<2	33 (76.7)	23 (79.3)	10 (71.4)	
≥2	10 (23.3)	6 (20.7)	4 (28.6)	
Liver inflammation grade, *n* (%)				0.307
<2	17 (39.5)	13 (44.8)	4 (28.6)	
≥2	26 (60.5)	16 (55.2)	10 (71.4)	
HBV DNA (log_10_ IU/mL)	2.35 (1.43–3.02)	2.19 (1.54–3.04)	2.39 (1.43–2.91)	0.948
HBsAg (log_10_ IU/mL)	1.92 (0.86–2.51)	1.92 (0.89–2.58)	1.91 (0.92–2.28)	0.808
HBsAg levels, *n* (%)				0.308
<200	15 (34.9)	12 (41.4)	3 (21.4)	
≥200	28 (65.1)	17 (58.6)	11 (78.6)	
HBeAg (C.O.I)	0.02 (0.00–0.21)	0.02 (0.00–0.20)	0.02 (0.00–0.21)	0.968
Anti-HBe (C.O.I)	0.01 (0.01–0.02)	0.01 (0.01–0.02)	0.01 (0.01–0.02)	0.580
ALT (U/L)	28 (19–58)	28 (19–45)	47 (21–77)	0.199
AST (U/L)	24 (19–33)	22 (18–32)	28 (19–42)	0.406
UA (mmol/L)	366 (307–420)	345 (246–408)	415 (355–474)	0.015
TC (mmol/L)	4.50 (4.00–5.35)	4.50 (3.90–5.15)	4.75 (4.10–5.86)	0.517
TG (mmol/L)	1.12 (0.75–1.52)	1.03 (0.69–1.30)	1.51 (0.84–1.69)	0.105
HDL (mmol/L)	1.35 (1.08–1.54)	1.35 (1.09–1.57)	1.33 (1.08–1.47)	0.509
LDL (mmol/L)	2.82 (2.28–3.55)	2.79 (2.23–3.34)	2.98 (2.34–3.81)	0.595
GLU (mmol/L)	5.16 (4.90–5.45)	5.16 (4.90–5.50)	5.12 (5.00–5.30)	0.979
TBIL (mmol/L)	12 (9–18)	12 (9–17)	15 (10–19)	0.351
DBIL (mmol/L)	4.40 (3.25–6.10)	3.70 (3.30–5.10)	4.75 (3.30–8.38)	0.259
ALP (U/L)	68 (55–78)	62 (51–70)	75 (68–83)	0.022
GGT (U/L)	21 (16–40)	19 (15–30)	32 (18–53)	0.126
Albumin (g/L)	46.00 (43.00–47.00)	45.00 (43.00–47.00)	47.00 (45.25–47.75)	0.117
Globulin (g/L)	29 (26–32)	27 (25–30)	31 (27–33)	0.104

^*a*^ Continuous variables are presented as medians (interquartile ranges).

^*b*^ *P *values are derived from Wilcoxon rank sum test, Pearson’s Chi-squared test, or Fisher’s exact test, as appropriate.

We further performed a multivariable Cox regression analysis with an interaction term (NAFLD × HBeAg) to explore whether the effect of NAFLD on HBsAg loss differed according to HBeAg status. The analysis revealed no significant difference in the impact of NAFLD on HBsAg loss according to HBeAg status (HR = 0.27, 95% CI: 0.01–5.07, *P* = 0.38) (Table 5). This finding further supports that among CHB patients receiving PegIFNα-based therapy, concomitant NAFLD does not significantly affect key outcomes such as HBsAg seroclearance or virologic suppression. Baseline HBsAg level remains a critical independent predictor of HBsAg clearance with PegIFNα therapy.

**TABLE 5:** Interaction analysis of NAFLD and HBeAg status on HBsAg clearance

Characteristics	HR (95% CI)^*a*^	*P* value
Age (years)	0.97 (0.93–1.02)	0.27
Gender, *n* (%)		
Male	–^*b*^	
Female	0.95 (0.36–2.52)	0.92
BMI classification		
Normal, [18.5, 23)	–	
Underweight, <18.5	1.13 (0.32–4.04)	0.85
Overweight, ≥23	0.63 (0.30–1.36)	0.24
UA (mmol/L)	1.00 (1.00–1.01)	0.62
TC (mmol/L)	0.76 (0.53–1.09)	0.13
TG (mmol/L)	0.62 (0.35–1.12)	0.12
Treatment regimen, *n* (%)		
PegIFNα	–	
PegIFNα + NAs	1.25 (0.62–2.51)	0.53
Liver fibrosis stage, *n* (%)		
<2	–	
≥2	1.23 (0.51–2.95)	0.64
Liver inflammation grade, *n* (%)		
<2	–	
≥2	0.83 (0.35–1.95)	0.67
Presence of fatty liver		
CHB	–	
CHB + NAFLD	3.75 (0.21–65.9)	0.37
HBeAg status		
Positive	–	
Negative	3.73 (0.39–35.8)	0.25
Baseline HBsAg (log_10_ IU/mL)	0.35 (0.24–0.52)	<0.001
Baseline HBV DNA (log_10_ IU/mL)	0.83 (0.62–1.10)	0.19
Baseline ALT (U/L)	1.00 (1.00–1.01)	0.79
ALP (U/L)	1.00 (0.98–1.02)	0.95
NAFLD × HBeAg interaction term		
CHB + NAFLD × HBeAg-negative	0.27 (0.01–5.07)	0.38

^*a*^ HR, hazard ratio; CI, confidence interval.

^*b*^ "–" denotes the reference group for the categorical variables in the Cox regression analysis. The hazard ratio (HR) for the reference group is fixed at 1.00; therefore, no confidence interval or *P* value is calculated.

To assess whether the predictive value of baseline HBsAg and HBV DNA for HBsAg clearance was influenced by the treatment regimen, we further performed a stratified analysis by treatment group (PegIFNα monotherapy vs PegIFNα + NAs). In the univariate analysis, baseline HBsAg and HBV DNA were both significant predictors of HBsAg clearance in both the PegIFNα monotherapy and combination therapy groups (*P* ≤ 0.05) (Table 6), suggesting that lower baseline viral levels are beneficial for achieving HBsAg clearance. In multivariable Cox regression analyses, the baseline HBsAg level remained a significant predictor of HBsAg clearance in both treatment groups (*P* ≤ 0.05). In contrast, HBV DNA lost its statistical significance after multivariable adjustment (Tables 7 and 8).

**TABLE 6:** Univariate Cox regression analysis for HBsAg clearance stratified by treatment group

Variable	PegIFNα (*n* = 80)		PegIFNα + NAs (*n* = 121)	
	HR (95% CI)^*a*^	*P* value	HR (95% CI)^*a*^	*P* value
Age (years)	0.99 (0.94–1.04)	0.62	1.06 (1.01-1.11)	0.019
Gender, *n* (%)				
Male	–^*b*^		–	
Female	1.69 (0.69–4.15)	0.25	0.96 (0.37–2.48)	0.94
Family history of hepatitis B, *n* (%)				
No	–		–	
Yes	0.17 (0.02–1.25)	0.082	0.66 (0.24–1.80)	0.42
Family history of liver cancer				
No	–		–	
Yes	1.08 (0.37–3.19)	0.89	0.97 (0.35–2.64)	0.95
Alcohol consumption history, *n* (%)				0.004
No	172 (85.6)	114 (91.2)	58 (76.3)	
Yes	29 (14.4)	11 (8.8)	18 (23.7)	
BMI (kg/m^2^)	23.3 (21.4–25.4)	22.0 (20.0–23.9)	25.1 (23.5–27.8)	<0.001
Underweight, [18.5, 23)	–		–	
Normal, <18.5	2.07 (0.56–7.64)	0.28	0.71 (0.09–5.59)	0.74
Overweight, ≥23	0.63 (0.26–1.55)	0.31	0.98 (0.41–2.36)	0.96
Liver fibrosis stage, *n* (%)				
<2	–		–	
≥2	1.16 (0.43–3.13)	0.78	1.02 (0.37–2.78)	0.97
Liver inflammation grade, *n* (%)				0.161
<2	–		–	
≥2	0.46 (0.19–1.09)	0.078	0.31 (0.13–0.74)	0.008
Steatosis grade				
<1	–		–	
≥1	0.74 (0.31–1.77)	0.50	0.77 (0.32–1.83)	0.55
Presence of fatty liver				
CHB	–		–	
CHB + NAFLD	0.75 (0.30–1.93)	0.56	0.84 (0.35–2.02)	0.69
HBV DNA (log_10_ IU/mL)	0.57 (0.44–0.74)	<0.001	0.58 (0.46–0.74)	<0.001
HBsAg (log_10_ IU/mL)	0.45 (0.34–0.60)	<0.001	0.22 (0.15–0.32)	<0.001
HBeAg (C.O.I)	0 (0–409)	0 (0–309)	0 (0–606)	0.877
Positive	–		–	
Negative	9.19 (1.24–68.4)	0.030	24.2 (3.25–181)	0.002
Anti-HBe (C.O.I)	0 (0–14)	1 (0–13)	0 (0–17)	0.373
Positive	–		–	
Negative	2.56 (0.76–8.66)	0.13	12.8 (2.98–55.1)	<0.001
ALT (U/L)	0.99 (0.99–1.00)	0.13	0.98 (0.97–1.00)	0.017
AST (U/L)	0.99 (0.98–1.00)	0.14	0.97 (0.95–1.00)	0.029
UA (mmol/L)	1.00 (1.00–1.01)	0.94	1.00 (1.00–1.01)	0.73
TC (mmol/L)	0.91 (0.58–1.41)	0.67	0.73 (0.44–1.21)	0.23
TG (mmol/L)	0.94 (0.50–1.75)	0.84	0.88 (0.53–1.47)	0.63
HDL (mmol/L)	0.95 (0.27–3.32)	0.93	1.54 (0.39–6.12)	0.54
LDL (mmol/L)	1.13 (0.73–1.73)	0.59	0.80 (0.47–1.34)	0.39
GLU (mmol/L)	2.00 (0.94–4.26)	0.072	0.99 (0.94–1.05)	0.78
TBIL (mmol/L)	0.95 (0.88–1.02)	0.16	1.00 (0.94–1.06)	0.96
DBIL (mmol/L)	0.81 (0.67–0.98)	0.034	0.94 (0.79–1.12)	0.49
ALP (U/L)	0.99 (0.97–1.01)	0.31	0.99 (0.97–1.01)	0.32
GGT (U/L)	0.99 (0.98–1.01)	0.33	0.97 (0.95–1.00)	0.032
Albumin (g/L)	1.05 (1.00–1.10)	0.036	1.06 (0.97–1.14)	0.19
Globulin (g/L)	1.02 (0.99–1.05)	0.18	0.94 (0.89–1.00)	0.061

^*a*^ HR, hazard ratio; CI, confidence interval.

^*b*^ "–" denotes the reference group for the categorical variables in the Cox regression analysis. The hazard ratio (HR) for the reference group is fixed at 1.00; therefore, no confidence interval or *P* value is calculated.

**TABLE 7:** Multivariate Cox regression analysis for HBsAg clearance stratified by treatment group (Model 1)

Variable	PegIFNα (*n* = 80)		PegIFNα + NAs (*n* = 121)	
	HR (95% CI)^*a*^	*P* value	HR (95% CI)^*a*^	*P* value
Liver fibrosis stage, *n* (%)				
<2	–^*b*^		–	
≥2	0.34 (0.04–2.95)	0.33	1.27 (0.42–3.87)	0.67
Liver inflammation grade, *n* (%)				
<2	–		–	
≥2	3.16 (0.91–11.0)	0.071	1.02 (0.26–3.95)	0.98
HBsAg (log_10_ IU/mL)	0.44 (0.26–0.73)	0.002	0.24 (0.14–0.43)	<0.001
HBV DNA (log_10_ IU/mL)	1.04 (0.63–1.71)	0.89	0.85 (0.57–1.28)	0.44
HBeAg (C.O.I)				
Positive	–		–	
Negative	5.84 (0.29–118)	0.25	1.52 (0.08–28.6)	0.78
Anti-HBe (C.O.I)				
Positive	–		–	
Negative	0.25 (0.05–1.28)	0.10	1.10 (0.12–9.79)	0.93
ALT (U/L)	1.00 (0.99–1.01)	0.71	1.00 (0.99–1.01)	0.94
DBIL (mmol/L)	0.87 (0.70–1.07)	0.18	1.04 (0.86–1.26)	0.70
GGT (U/L)	0.99 (0.97–1.01)	0.35	0.99 (0.96–1.01)	0.40
Albumin (g/L)	1.02 (0.97–1.08)	0.36	1.05 (0.95–1.15)	0.33

^*a*^ HR, hazard ratio; CI, confidence interval.

^*b*^ "–" denotes the reference group for the categorical variables in the Cox regression analysis. The hazard ratio (HR) for the reference group is fixed at 1.00; therefore, no confidence interval or *P* value is calculated.

**TABLE 8:** Multivariate Cox regression analysis for HBsAg clearance stratified by treatment group (Model 2)

Variable	PegIFNα (*n* = 80)		PegIFNα + NAs (*n* = 121)	
	HR (95% CI)^*a*^	*P* value	HR (95% CI)^*a*^	*P* value
HBsAg (log_10_ IU/mL)	0.61 (0.40–0.93)	0.022	0.27 (0.17–0.41)	<0.001
HBV DNA (log_10_ IU/mL)	0.74 (0.53–1.04)	0.087	0.79 (0.57–1.10)	0.17

^*a*^ HR, hazard ratio; CI, confidence interval.

## DISCUSSION

This study conducted a multidimensional assessment to determine whether concurrent NAFLD comorbidity influences clinical outcomes in CHB patients receiving PegIFNα-based therapy, revealing selective effects—primarily on the biochemical response, and not on virological outcomes or HBsAg clearance. At week 48, patients with CHB + NAFLD exhibited significantly lower biochemical response rates compared to those with CHB alone. This finding aligns with previous reports suggesting that NAFLD reduces the biochemical response in CHB patients (14, 15). While ALT elevation in CHB-only cases is primarily HBV-driven and typically resolves with viral suppression, NAFLD independently contributes to persistent mild-to-moderate ALT elevation through metabolic and inflammatory hepatocellular injury (16, 17), even after HBV replication is controlled. This persistent ALT elevation compromises the normalization threshold, thereby directly reducing the biochemical response rate in patients undergoing PegIFNα treatment.

Crucially, regarding our primary focus on the PegIFNα response, concurrent NAFLD did not significantly influence HBsAg clearance, which represents the optimal therapeutic endpoint and a key goal of PegIFNα therapy. Kaplan-Meier survival curves revealed comparable time-to-clearance patterns between CHB patients with and without NAFLD. Multivariable Cox regression analysis specifically identified baseline HBsAg and HBV DNA levels, but not NAFLD status, as independent predictors of HBsAg seroclearance. This finding in the context of PegIFNα treatment contrasts with some studies suggesting that NAFLD may facilitate HBsAg clearance via immune modulation or indirect viral suppression (18, 19). However, it aligns with other studies reporting no significant association, or even potential adverse impacts due to NAFLD-related fibrosis progression (20, 21). Subgroup analysis of patients achieving HBsAg clearance showed nearly identical baseline HBsAg levels (median: 1.92 log_10_ IU/mL in CHB + NAFLD vs 1.91 log_10_ IU/mL in CHB-only), strongly suggesting that the baseline HBsAg burden plays a more decisive role in HBsAg clearance during PegIFNα therapy than the presence of NAFLD.

Similarly, NAFLD had no significant impact on virologic response rates in CHB patients treated with PegIFNα-based regimens. This aligns with several prior studies reporting a minimal effect of NAFLD on PegIFNα efficacy (22, 23), diverging from other literature indicating a diminished response in NAFLD cases (11, 13, 24). In our cohort, the use of combination therapy—PegIFNα and NAs—may have obscured subtle NAFLD-related effects on virological suppression. Variations in demographic features and baseline virologic profiles—including age, HBV DNA, and HBsAg levels—could directly affect the difficulty of attaining HBsAg clearance. Furthermore, the severity of NAFLD varied across studies. Compared to simple steatosis, steatohepatitis with significant inflammatory activity may exert a stronger interference with the immunomodulatory effects of interferon, thereby having a greater impact on HBsAg clearance.

Notably, studies on PegIFNα monotherapy have reported a reduced response in NAFLD patients, potentially due to obesity-related changes in interferon pharmacokinetics and signaling dynamics (25–27). Consequently, therapeutic decisions for CHB with concurrent NAFLD should integrate both virologic and metabolic profiles, tailoring regimens based on disease characteristics and anticipated treatment responses. PegIFNα remains a viable option for achieving HBsAg clearance, especially in patients with favorable baseline predictors. These patients are also eligible to pursue the goal of a clinical cure for hepatitis B. However, baseline HBsAg and HBV DNA levels should be carefully assessed before treatment to more accurately predict the likelihood of HBsAg clearance.

Our multivariate analysis confirmed the baseline HBsAg level as an independent predictor of HBsAg clearance, aligning with prior research (28). A low baseline HBsAg level is thus a key favorable condition for achieving a functional cure in CHB patients with PegIFNα. Additionally, the baseline HBV DNA level was identified as another independent negative predictor. Reduced viral replication increases the likelihood of HBsAg clearance. Notably, univariate predictors (e.g., hepatitis B family history and HBeAg status) lost statistical significance in the multivariate models, suggesting that their effects are mediated through or confounded by HBsAg and HBV DNA levels. Thus, baseline quantitative measurements of HBsAg and HBV DNA should be prioritized in predicting and guiding PegIFNα-based management strategies aimed at HBsAg clearance.

Our study found that although NAFLD does not significantly impair HBsAg clearance, it was associated with reduced biochemical response rates. Therefore, the management of hepatic steatosis and associated metabolic comorbidities remains critical in patients with CHB and NAFLD. This requires comprehensive lifestyle interventions, including weight control, dietary adjustments, and increased physical activity, along with the pharmacologic control of metabolic risk factors such as hyperglycemia, insulin resistance, and dyslipidemia. These strategies not only improve hepatic function and potentially enhance the biochemical response during antiviral therapy but may also reduce the long-term HCC risk and enhance overall liver health outcomes, complementing the antiviral effects of PegIFNα (29). Due to the low biochemical response rate, close monitoring of liver function, particularly changes in aminotransferase levels, should be intensified during PegIFNα therapy to facilitate the timely identification and management of potential hepatic inflammation.

Despite its strengths, this study has limitations. As a single-center retrospective study with a relatively small sample size, the generalizability of the findings is limited. Future multicenter prospective cohort studies with larger sample sizes are necessary to produce higher-level evidence-based conclusions. In addition, a more detailed integration of liver histology data—such as quantifying the degree of steatosis, inflammation (NASH), and fibrosis—in future studies would more accurately delineate the spectrum of NAFLD and its specific impact on the PegIFNα treatment response. Furthermore, the exploration of molecular mechanisms underlying NAFLD-HBV interactions in the context of interferon signaling is warranted, particularly their influence on host immune responses, including T cell function and cytokine networks, viral replication cycles, and the intrahepatic microenvironment. These insights may provide a theoretical foundation for novel therapeutic approaches targeting CHB patients with concurrent NAFLD.

### Conclusions

In summary, for CHB patients undergoing PegIFNα-based therapy, concurrent NAFLD does not impair the crucial outcomes of HBsAg seroclearance or virological suppression but is associated with a reduced biochemical response rate. Baseline HBsAg and HBV DNA levels serve as key independent predictors of HBsAg clearance with PegIFNα and should be emphasized in clinical evaluation and treatment planning. Therefore, optimal clinical management of CHB patients with NAFLD should integrate both antiviral strategies and metabolic control. Regular monitoring of virological markers—particularly quantitative HBsAg—alongside the active management of steatosis and metabolic risk factors, is essential to improve hepatic outcomes and long-term prognosis.
